# Oxidative stress in the brain and retina after traumatic injury

**DOI:** 10.3389/fnins.2023.1021152

**Published:** 2023-02-03

**Authors:** Annie K. Ryan, Wade Rich, Matthew A. Reilly

**Affiliations:** ^1^Department of Biomedical Engineering, The Ohio State University, Columbus, OH, United States; ^2^Department of Ophthalmology and Visual Sciences, The Ohio State University, Columbus, OH, United States

**Keywords:** trauma, optic neuropathy, traumatic brain injury, ROS, neurodegeneration

## Abstract

The brain and the retina share many physiological similarities, which allows the retina to serve as a model of CNS disease and disorder. In instances of trauma, the eye can even indicate damage to the brain *via* abnormalities observed such as irregularities in pupillary reflexes in suspected traumatic brain injury (TBI) patients. Elevation of reactive oxygen species (ROS) has been observed in neurodegenerative disorders and in both traumatic optic neuropathy (TON) and in TBI. In a healthy system, ROS play a pivotal role in cellular communication, but in neurodegenerative diseases and post-trauma instances, ROS elevation can exacerbate neurodegeneration in both the brain and the retina. Increased ROS can overwhelm the inherent antioxidant systems which are regulated *via* mitochondrial processes. The overabundance of ROS can lead to protein, DNA, and other forms of cellular damage which ultimately result in apoptosis. Even though elevated ROS have been observed to be a major cause in the neurodegeneration observed after TON and TBI, many antioxidants therapeutic strategies fail. In order to understand why these therapeutic approaches fail further research into the direct injury cascades must be conducted. Additional therapeutic approaches such as therapeutics capable of anti-inflammatory properties and suppression of other neurodegenerative processes may be needed for the treatment of TON, TBI, and neurodegenerative diseases.

## 1. Introduction

**FIGURE 1:**
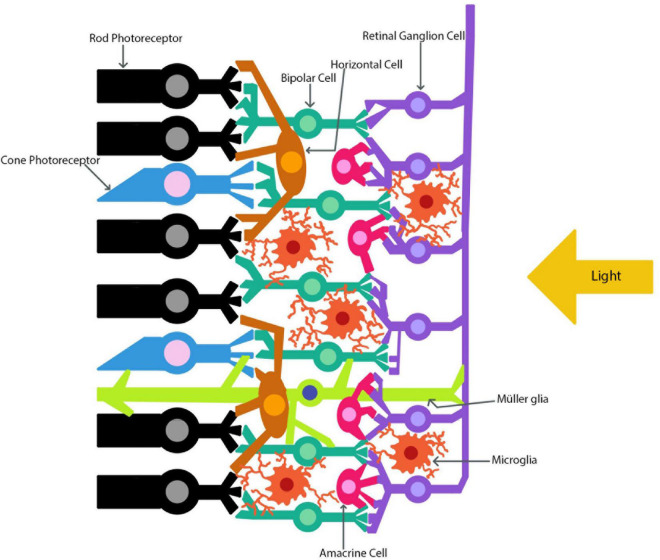
The retinal layers. The path of light as it travels through the retina to the photoreceptors is indicated. The retinal ganglion cell axons combine to form the optic nerve.

The eye and the brain are closely connected and thus share many similarities. Many researchers have focused on studying ocular phenomena *via* examination of the retina since the eye is much more accessible and yet shares many of the same physiological, anatomical, and developmental characteristics as the brain (Cheung et al., 2017; Bernardo-Colón et al., 2018). The eye allows for close observation of the central nervous system (CNS) as both the neurons and blood vessels can be directly monitored (Nguyen et al., 2021). As Ptito et al. (2021) state, “no other part of the central nervous system is amenable to direct observation” (2021, p. 7). This unique relationship between the eye and the brain allows the eye to serve as a model for CNS disease. Also, many neurodegenerative diseases such as Alzheimer’s Disease (AD), Parkinson’s Disease (PD), and Multiple Sclerosis (MS) present with changes in ocular physiology (Blanks et al., 1989; Archibald et al., 2009; Green et al., 2010; Lee et al., 2020; Nguyen et al., 2021). The eye is often referred to as an extension of the brain, and this can be exemplified through the optic nerve developmental process. By examining how the retina develops one can see how interlinked the brain and the retina are. Figure 1 depicts the retinal layers with the RGC axons joining to form the optic nerve. During development, the retinal ganglion cell (RGC) axons begin to extend through the optic nerve and toward the brain (Nickells, 1996). Once the RGC axons reach the brain they then form synaptic connections with their respective target sites before uptaking brain-derived neurotrophic factor (BDNF), which is produced by neuronal cells (Nickells, 1996; Brooks et al., 1999). Before this point, developing RGCs did not need BDNF to survive, but once the first synaptic connection is made between the brain and the retina, BDNF is transported to the retina (Nickells, 1996; Brooks et al., 1999). From this developmental point onward RGCs will need BDNF in order to survive (Nickells, 1996; Brooks et al., 1999). The brain and retina also share high concentrations of the same forms of neurotransmitters, such as glutamate (Nickells, 1996; Wu and Maple, 1998). Both the brain and retina have high physiological energy requirements, which require many mitochondria in order to produce the necessary energy requirements (Rango and Bresolin, 2018; Eells, 2019; Singh et al., 2019; Nascimento-dos-Santos et al., 2020). As well as providing energy, mitochondria also help to regulate other essential cellular functions such as apoptosis, reactive oxygen species (ROS) production, antioxidant regulation *via* the mitochondrial thioredoxin system, intracellular calcium regulation, and many other roles (Forred et al., 2017; Angelova and Abramov, 2018; Robicsek et al., 2018). Mitochondria serve a crucial role in maintaining homeostatic conditions in cells, and thus, when they function irregularly, disastrous consequences arise. Dysfunctions in mitochondria have been linked to several neurodegenerative diseases in both the brain and the retina, which can lead to over production of ROS (Angelova and Abramov, 2018; Singh et al., 2019; Nascimento-dos-Santos et al., 2020).

The means in which trauma presents to the brain can also be translated to the eye. Traumatic brain injury (TBI) develops after direct or indirect trauma to the brain and can present as changes in consciousness, impaired cognitive ability, memory deficits, changes in vision, and even death (Rodgers et al., 2012; Mohan et al., 2013; Ohta et al., 2013; Brooks et al., 2014; Rubiano et al., 2015; Galgano et al., 2017; Gupta et al., 2019). Traumatic optic neuropathy (TON) is an irreversible vision-loss condition that is often developed after TBI (Sarkies, 2004; Sherwood et al., 2014). Similar to TBI, TON can develop after either direct or indirect injury. Direct TON (dTON) develops when the optic nerve is directly impacted (e.g., optic nerve crush or severing), while indirect TON (iTON) develops as a result of blunt force injury and the neuroinflammatory process that arises afterward (Sarkies, 2004; DeJulius et al., 2021).

The eye can be used to glean information about the brain. For example, the eye has been used screen for potential TBIs by medical professionals for years through the observation of pupillary reflexes (Adoni and McNett, 2007). One of the first tests done to a suspected TBI patient is to shine a light into their eyes and note if there are any abnormalities in the way their pupils respond to the light. An abnormal pupillary response is linked to TBI (Adoni and McNett, 2007).

The brain and retina also share a similar mechanism in order to protect themselves known as the blood-brain and blood-retina barrier (BBB and BRB) (Streit et al., 2004; Bond and Rex, 2014). As such, these barriers keep unwanted cells and other pathogens out of the CNS, but if these barriers are disrupted, as is the case in injury, then immune cells can migrate into both the brain and retina (Streit et al., 2004; Bond and Rex, 2014; DeJulius et al., 2021). Macroglia, such as astrocytes, are important glial cells responsible for maintenance of the BBB (Cabezas et al., 2014). The mature brain and retina both contain astrocytes and microglial cells (Kolb, 2001; Purves et al., 2001). One form of macroglia that is unique to the brain is the oligodendrocytes, which serve to build myelin around certain axons (Purves et al., 2001). The retina also has a unique macroglia, which is known as the Müller glia and is depicted in Figure 1 (Kolb, 2001). The Müller glial cells are the main glial cell of the retina and help to maintain homeostasis while providing environmental protection (Kolb, 2001).

The damage mechanisms in TBI and traumatic optic neuropathy (TON) are not fully understood; however, it is known that their neuroinflammatory processes tend to occur secondary to initial injury (Rodgers et al., 2012; Zhou et al., 2021; Priester et al., 2022). It has also been concluded that ROS play a major role in the observed neurodegeneration after TON and TBI (Bernardo-Colón et al., 2018; DeJulius et al., 2021; Priester et al., 2022). The specific pathways in which traumatic injury initiates neurodegeneration and how increased levels of ROS are produced are not fully understood. In addition, the means to treat neurodegeneration after TBI and TON remain elusive with many attempts at antioxidant therapeutics ultimately failing at the clinical level (Angelova and Abramov, 2018; Priester et al., 2022). In order to determine efficacious therapeutics, we must first determine the means in which neurodegeneration progresses after injury.

## 2. The role of ROS in the healthy brain and retina

Reactive oxygen species are a group of unstable molecules which are produced under normal physiologic conditions through the partial reduction of molecular oxygen (Hsieh et al., 2014; Nita and Grzybowski, 2016; Sies and Jones, 2020; Yang and Lian, 2020). Such molecules include hydroxyl radical (OH), superoxide (O_2_^–^), hydrogen peroxide (H_2_O_2_), and singlet oxygen (^1^O_2_) (Hsieh et al., 2014; Sies and Jones, 2020; Yang and Lian, 2020). Reactive nitrogen species (RNS) also play a role as signaling molecules and in unbalanced systems can result in disease states (Hsieh et al., 2014). Common examples of RNS include nitric oxide (NO), peroxynitrite (OONO^–^), and nitrogen dioxide (NO_2_^–^) (Hsieh et al., 2014). Under normal physiological conditions NO can be produced *via* shear stress and plays an important role in the regulation of vasculature (Metea and Newman, 2006; Attwell et al., 2010; Mishra and Newman, 2010; Hsieh et al., 2014; Someya et al., 2019). ROS in small concentrations are beneficial and highly necessary for daily functioning in cells (Dröge, 2002) as signaling molecules and are involved in feedback inhibition loops, which help to maintain redox balance within cells (Angelova and Abramov, 2018). In neuronal cells ROS play a vital role in neuronal differentiation through neuronal growth factor (NGF) induced differentiation (Suzukawa et al., 2000). The functionality of ROS as a beneficial signaling agent at low concentrations and damaging oxidative stressor at high concentrations appears to be present systemically, and certainly can be found within the brain and retina. One study investigating diabetic retinopathy concluded that ROS generation in retinal cells is regulated by glucose concentration in a concentration dependent manner (Zhang et al., 2019). Low levels of glucose led to retinal pigment epithelium mitophagy, which could have potentially protective effects, with no impact to cell proliferation or apoptosis, while high levels of glucose inhibited cell proliferation, induced apoptosis, and initiated ROS mediated gene inactivation (Zhang et al., 2019).

Healthy mitochondria play an important role in helping to maintain redox homeostasis balance in cells *via* the mitochondrial thioredoxin system and are usually protected by this system (Forred et al., 2017; Angelova and Abramov, 2018). The mitochondria is a large producer of ROS as it is a by-product of ATP synthase (Yang and Lian, 2020). Scortegagna et al. (2003) examined the relationship between hypoxia inducible factor-2 alpha (HIF-2α), antioxidant enzymes, and ROS concentrations. Their results suggest ROS regulation in the mitochondria may be partially controlled by HIF-2α (Scortegagna et al., 2003). Other enzymes and antioxidants for downregulation of ROS include vitamins E and C, superoxide dismutase 2 (SOD2), and glutathione peroxidase 1 (GPx1) (Bernardo-Colón et al., 2018; Yang and Lian, 2020). If these antioxidants fail at maintaining homeostatic concentrations of ROS, then deleterious effects can be observed. While the mitochondria is a large producer of ROS, it can also become the target of ROS damage (Angelova and Abramov, 2018). One group observed that an increase in mitochondrial ROS was triggered by excess levels of tumor necrosis factor (TNF) (Roca and Ramakrishnan, 2013). The mitochondria can also experience ROS-induced ROS release (Hiebert et al., 2015). Under normal conditions, the mitochondria can maintain ROS concentrations through the regulation of mitochondrial permeability transition pore (mPTP) openings (Hiebert et al., 2015). However, when ROS levels become too high for the mPTP openings to sufficiently regulate the levels, and the antioxidant enzymes become overwhelmed, the mitochondria can then release a burst of ROS (Hiebert et al., 2015). This excessive release of ROS damages the mitochondria, and potentially neighboring mitochondria, and leads to a decrease in ATP synthesis and increase in apoptotic processes (Hiebert et al., 2015). In a rodent TBI model, it was observed that mitochondrial bioenergetics in the form of respiration are significantly decreased after injury as compared to sham control animals (Pandya et al., 2014). Sullivan et al. (2004) described how the change in ATP production after TBI may arise. They summarize that after TBI calcium levels rise, followed by mitochondrial channel openings increasing to allow an increased flux of calcium into the mitochondrion (Sullivan et al., 2004). This then destabilizes the electron transport chain which is followed by a decrease in ATP production, mitochondrial membrane potential, and increased levels of ROS (Sullivan et al., 2004). During this process, the mitochondria swell, which results in the release of proapoptotic proteins. In summary, when the mitochondria become the site of ROS-induced damage, it fails to function properly, which can cause unwanted affects to the mitochondria’s role of energy production.

Nitric oxide can also be beneficial or detrimental depending on concentration. While low levels of NO are necessary for signaling, higher concentrations can be harmful to neurons (Singh et al., 2019; Mueller-Buehl et al., 2021). In the retina, small concentrations of NO are utilized for light adaptation, visual processing, and amplification of visual responses in various retinal cell types (Vielma et al., 2012; Mueller-Buehl et al., 2021). NO can be produced by peroxisomes as well as other sources and is an indispensable messenger molecule responsible for regulating vascular tone, blood clotting, inflammation, and serving as a cardiovascular protectant (Naseem, 2005). Both the brain and the retina utilize NO signaling pathways to regulate vascular dilation and constriction (Metea and Newman, 2006; Someya et al., 2019). Someya et al. (2019) determined stimulated retinal neuronal cells release NO which then act as an important messenger to activate glial cell secretion of vasodilatory metabolites. Similar to Someya et al. (2019), Attwell et al. (2010) determined active neurons stimulate an increase in blood flow into their locations. They observed this phenomenon, which is termed hyperemia, and observed secreted glutamate triggers NMDA receptors which results in increased flux of calcium into neurons (Attwell et al., 2010). This increase of intracellular calcium then activates neuronal nitric oxide synthase (nNOS), which results in a higher concentration of NO which then leads to vasodilation (Attwell et al., 2010). NO can also regulate vasoconstriction as detailed by Metea and Newman (2006). They observed increased levels of NO resulted in greater vasoconstriction and decreased levels resulted in vasodilation (Metea and Newman, 2006). The authors do note that in general NO is considered a vasodilating agent; however, their results indicated NO promoted vasoconstriction in the retina (Metea and Newman, 2006). Mishra and Newman (2010) also determined that glial cell and light stimulation can induce vasoconstrictions through elevated NO.

Reactive oxygen species found in peroxisomes assist animal cells with the critical function of fatty acid oxidation which allows for metabolic energy release. Peroxisomes also utilize ROS during the synthesis of lipids such as cholesterol (Cooper, 2000). Peroxisomes, originally thought to be merely a sink for excess cellular hydrogen peroxide, are now known to be involved in many complex metabolic pathways and are an essential source of reactive nitrogen species (RNS) including NO. Oversaturation of ROS and RNS can lead to chemical stress, but low concentrations such as those found in healthy peroxisomes are essential for cellular signaling. One study found that peroxisomes can sense and respond to environmental cues from ROS and redox changes and play a key role in maintaining redox homeostasis (Sandalio and Romero-Puertas, 2015). The study demonstrated that signaling pathways involving peroxisomes both sensed ROS change in the environment and responded by manipulating target genes involved in the cellular response to oxidative stress. Peroxisomes were also found to be involved in hormone production (Sandalio and Romero-Puertas, 2015). Another study found that peroxisomes are present and active within the murine retina (Das et al., 2019). Significantly, different retinal cells expressed different levels of peroxisome activity suggesting that peroxisomes may have unique roles within different retinal tissues (Das et al., 2019).

Another organelle which utilizes ROS is the lysosome. Lysosomes are required for cellular digestive processes, waste removal, and molecular scavenging from damaged or outdated cellular matter. Similarly, to the peroxisome, lysosomes are involved in metabolic signaling and require a low concentration of ROS to perform their cellular function (Lim and Zoncu, 2016). High levels of ROS, however, have been shown to inhibit lysosomal activity, reduce lysosomal motility, and prevent lysosomal fusion with target molecules (Saffi et al., 2021). Lysosomes are also active in the retina and assist with regulation of autophagy. Autophagy is known to decrease with age which can result in accumulation of waste or a decrease to cellular organization. This age associated reduction to beneficial autophagy, essentially acting as cellular cleaning, may be linked to development of disease such as age associated macular degeneration (Sinha et al., 2016). Lysosomal activity and autophagy with associated ROS is also linked with synaptic pruning in the brain (Song et al., 2008).

Peroxisomes and lysosomes are present in both the retinal cells and brain cells. In the brain, peroxisomes have been detected in all neural cell types and specifically measured in neurons, oligodendrocytes, astrocytes, microglia, and endothelial cells (Berger et al., 2016). Within the brain peroxisomes appear as single membrane-bound organelles and are smaller than peroxisomes found in other tissues (Berger et al., 2016). Peroxisomes contribute to lipid metabolism, and are membrane associated in neural cells, so healthy peroxisomal function is crucial for proper development and health of myelin sheaths in the brain white matter (Kassmann, 2014). Lysosomes are also found within neurons including pyramidal cell, mitral cell, hippocampal granule, and olfactory bulb neurons (Roberts and Gorenstein, 1987). Lysosomal distribution within the neuron; clustering within the dendrites, axon, or cell body; was shown to fluctuate across the lifespan of an organism as well (Roberts and Gorenstein, 1987; Ferguson, 2019). Lysosomes are present in glial cells and assist with glial functions such as molecular secretion, uptake, and degradation in astrocytes, oligodendrocytes, and microglia (Kreher et al., 2021). Peroxisomes have been detected within every retinal cell layer, although their distribution is not uniform (Das et al., 2019). Lysosomes have been studied within retinal pigmented epithelial cells (Sinha et al., 2016), linked to photoreceptor cell homeostasis (Santo and Conte, 2021), and lysosomal localization in the retinal pigmented epithelia linked to downstream photoreceptor health (el-Hifnawi et al., 1994). Within the retina, lysosomes appear to be most concentrated in the retinal pigmented epithelia, and are found within photoreceptors (Strauss, 2005). Lysosomal function within the retinal pigmented epithelia can have downstream effects on the neuronal retina and photoreceptor cells (Strauss, 2005).

It is evident that ROS are ubiquitous throughout the body and hold critical roles in cellular signaling, metabolism, digestion, organization, and energy economics. ROS are found in the healthy retina and brain carrying out similar functions as they do throughout the body. There also appears to be a nearly universal trend exhibited by ROS in the body in which their effects on cellular function switch from supportive to harmful as ROS concentration increases. These species are necessary in low concentrations for healthy tissue function, but in large doses can induce oxidative stress or damage tissues. The homeostatic balance of ROS is vital to proper cellular function. When focusing on ROS accumulation with age, it may seem at first that elimination of ROS and their damaging properties entirely would be desired. Under further scrutiny, however, it is apparent that the functionality of ROS at low concentrations is conductive to proper cell health.

## 3. Role of ROS in TBI, TON, and other neurodegenerative diseases

### 3.1. Traumatic brain injury (TBI)

Traumatic brain injury is one of the leading forms of death as a result of trauma related injury (Rubiano et al., 2015; Priester et al., 2022). About 69 million people worldwide develop a TBI each year with children and young adults making up the majority of cases (Galgano et al., 2017; Dewan et al., 2018; Priester et al., 2022). TBI can result in death, changes in vision, impaired cognitive attention, issues with function, memory deficits, and changes in behavior (Rodgers et al., 2012; Mohan et al., 2013; Ohta et al., 2013; Brooks et al., 2014; Rubiano et al., 2015; Gupta et al., 2019). TBI has two parts: primary and secondary injury (Galgano et al., 2017; Tan et al., 2018; Priester et al., 2022). Primary injury occurs due to direct physical forces impacting the brain (Galgano et al., 2017; Tan et al., 2018; Priester et al., 2022). Secondary injury can occur several minutes to weeks after primary injury and encompasses cortical edema, BBB breakdown, ROS release, calcium imbalances, inflammatory cascades, and other cellular and molecular changes (Galgano et al., 2017; Priester et al., 2022). Post-TBI brains can develop swollen neurons, vacuolar changes, uncentered nuclei, and the loss of both white and gray matter (Sen, 2017; Tan et al., 2018). Tissue degeneration can be tracked through silver staining in brains (Deng et al., 2007). Deng et al. (2007) observed a significantly increased volume of silver staining with a maximal peak occurring at 48 h after injury. They observed the silver staining and subsequent neurodegeneration continued even at 7 days post injury (Deng et al., 2007). This is one example of how secondary TBI can exacerbate neurodegeneration even days after primary injury. After TBI, changes in ocular tissues can be observed as well. Models of TBI have reported changes in retinal nerve fiber layer (RNFL) thickness along with a decrease in oligodendrocyte precursor cells (OPCs) and subsequent reduction in myelination (Gupta et al., 2019). Currently there are no effective neuroprotective pharmaceutical therapeutics (Galgano et al., 2017; Priester et al., 2022).

### 3.2. Traumatic optic neuropathy (TON)

Eye related injuries encompass ∼2.5 million emergency department visits per year in the US alone, and account for 13% of battlefield injuries (Sherwood et al., 2014). TON is an irreversible vision-threatening complication of blunt force trauma, often affiliated with a TBI (Sarkies, 2004; Sherwood et al., 2014). TON is divided into two main categories: direct and indirect. Direct TON (dTON) occurs when the eye has been penetrated and encompasses injuries such as foreign bodies piercing the optic nerve, or orbital fractures crushing and/or severing the optic nerve (Sarkies, 2004). Indirect TON (iTON) is the more common form; however, it may not be detected as quickly (Sarkies, 2004). iTON is the result of blunt force trauma of the head, or TBI (Sarkies, 2004; DeJulius et al., 2021). About 0.5–5% of closed head TBIs result in TON (Sarkies, 2004). Currently, intervention relies on corticosteroids and/or surgical decompression (Sarkies, 2004). However, both have limited success and more importantly there is a growing concern of utilizing corticosteroids in the presence of TBI (Roberts et al., 2004; Sarkies, 2004; Steinsapir and Goldberg, 2011). In addition, translational animal models remain limited. TON is correlated with a deficit of RGCs and axon degeneration, but the exact disease progression and cell death pathways are not fully understood (Tse et al., 2018; Khan et al., 2021). ROS have been observed to increase while antioxidant enzymes, such as SOD, decrease after injury (Bernardo-Colón et al., 2018). Bernardo-Colón et al. (2018) also observed blast induced TON, caused vacuolization and hyper-myelination in optic nerves. Myelin injury, decreases in retinal nerve fiber layer thickness, and overall changes in retinal thickness have also been noted in TON models (Brooks et al., 2014; Jones et al., 2016; Evanson et al., 2018). It has also been noted that ROS plays a major effect in the degeneration of axons and subsequent vision loss after iTON (Bernardo-Colón et al., 2018; DeJulius et al., 2021).

### 3.3. ROS and the disease progression in TON and TBI

**FIGURE 2:**
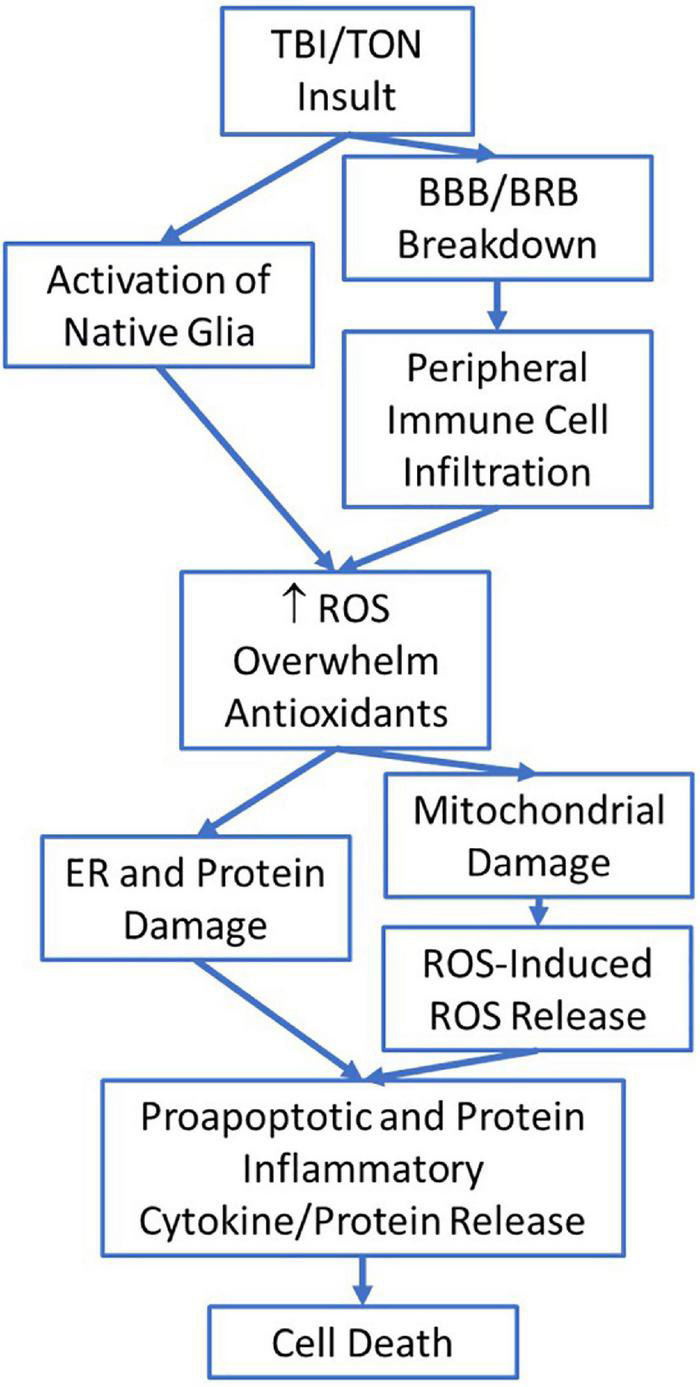
The potential flow of injury mechanisms and steps involved after traumatic brain injury (TBI)/traumatic optic neuropathy (TON) which eventually lead to cell death.

Like TBI, the vision loss associated with iTON is usually a secondary injury event (Tandon and Maapatra, 2017; Zhou et al., 2021). Li et al. (2020) conducted a study in which they modeled different blunt force head injuries to determine how iTON may be induced. From their study they determined a blunt force impact to the center of the forehead (0°) and a blunt force impact to the right frontal region of the forehead (45°) resulted in injury to the optic nerve *via* a shearing form of injury (Li et al., 2020). They determined blunt force impact to the head can result in stress wave propagation through the orbital rim to the optic canal (Li et al., 2020). In their model they observed the translation of impact energy propagated along the orbital ceiling to the top rim of the optic canal, which lead to optic canal diameter reduction (Li et al., 2020). This reduction may also play a role in facilitating secondary injury events, especially in the intracanalicular optic nerve as this area normally has limited space, and further reduction mixed with the start of neuroinflammatory responses could facilitate further compression of the optic nerve (Sarkies, 2004; Li et al., 2020). After the primary injury, secondary neurodegeneration occurs as neuroinflammation and ROS species accumulate near the site of injury (Figure 2) (Dröge, 2002; Bond and Rex, 2014). Before injury, the CNS and eye lack systemic macrophages due to their strict BBB/BRB, but they have microglia which act as immune cells and work to maintain the neurological tissue (Dröge, 2002; Bond and Rex, 2014; Tao et al., 2017; Thompson and Tsirka, 2017; McMenamin et al., 2019; DeJulius et al., 2021). After injury, the BBB/BRB can break down and allow for infiltration of peripheral immune cells such as macrophages, neutrophils, and leukocytes (Bond and Rex, 2014; Brooks et al., 2014; Galgano et al., 2017; McMenamin et al., 2019; DeJulius et al., 2021). As a result, ROS levels can become increasingly elevated as activated macrophages and other immune cells release large amounts of ROS when in inflammatory environments (Dröge, 2002). In TON it may be that RGC and axonal degeneration progress due to the breakdown of the BRB and subsequent infiltration of systemic immune cells which then produce large quantities of ROS. Once macrophages infiltrate the damaged CNS, they take on an active microglial morphology and begin to produce pro-inflammatory cytokines alongside the native active microglial cells (Bond and Rex, 2014; DeJulius et al., 2021). Elevated ROS may be too much for the mitochondria to combat *via* their redox homeostasis system, leading to mitochondrial damage and genetic alterations (Deng et al., 2007; Gupta et al., 2019).

Injury may cause mitochondrial dysfunction, which alters the mitochondrial redox homeostasis system, with respect to overproduction of ROS, and alterations of ROS-mediated gene expression (Dröge, 2002; Angelova and Abramov, 2018). When ROS is being overproduced by the mitochondria it can cause lipid peroxidation to start and in turn activate microglia (Angelova and Abramov, 2018). The dysfunction in the mitochondria limits the cells’ ability to combat the rising ROS with natural antioxidants, which then can lead to neurological degeneration and subsequent cell death (Angelova and Abramov, 2018). One study even observed irregularities in the shape of mitochondria after TBI (Tan et al., 2018). In this study, mitochondria in cortical neurons were observed to be swollen and misshaped with alterations and disruptions present in their cristae (Tan et al., 2018). Mueller-Buehl et al. (2021) also observed swollen mitochondria in their H_2_O_2_ induced retinal degeneration model. In addition, Tan et al. (2018) also examined endoplasmic reticulum (ER) stress and dysfunction in the context of TBI.

The ER is a large organelle in the cell and is responsible for several key functions including protein synthesis and transport, lipid synthesis, protein folding, steroid synthesis, and calcium storage (Schwarz and Blower, 2016). ER stress occurs when there is an accumulation of unfolded or misfolded proteins and as a result the unfolded protein response (UPR) is initiated to return the ER to normal function (Nakka et al., 2016; Schwarz and Blower, 2016; Tan et al., 2018). Activation of ER stress pathways have been observed to promote the formation of ROS through inducible nitric oxide synthase (iNOS)- dependent and -independent pathways (Hsieh et al., 2007). If function cannot be restored, then cell death and apoptosis occur (Schwarz and Blower, 2016). ER stress has also been observed in neurological cells after TBI (Deng et al., 2007; Logsdon et al., 2014, 2016; Hylin et al., 2018; Tan et al., 2018). Tan et al. (2018, p. 832) noted proteins related to ER stress were activated immediately after TBI and peaked 3 h after the injury. A separate study found ER stress related markers were increased 24 h after blast induced TBI (Logsdon et al., 2014). Similarly, Dash et al. (2015) observed significantly increased levels of eIF2α, a known marker of ER stress, at 24 h after injury in their rodent TBI model. Both Lucke-Wold et al. (2016) and Logsdon et al. (2016) noted an elevation in C/EBP homologous protein (CHOP) expression 24 h after their rodent blast injury model, indicating ER stress was induced. Lucke-Wold et al. (2016) also observed increased levels in human brain samples of patients inflicted with chronic traumatic encephalopathy (CTE), which further promotes ER stress’s role in neurodegeneration after neurotrauma (Lucke-Wold et al., 2016). Interestingly, Hylin et al. (2018) observed an increase in CHOP and binding immunoglobulin protein (BiP), another marker of ER stress, were significantly increased as early as 4 h after TBI in their juvenile rodent TBI model. Tan et al. (2018, p. 833) found that ER were abnormally shaped and swollen in cortical neurons after TBI. This same study observed the apoptotic ER stress pathway was maximally activated 6 h after injury followed by activation of the mitochondrial apoptotic pathway, which occurred 6 h after TBI (Tan et al., 2018). Tan et al. (2018, p. 835) determined that inhibition of ER stress *via* the ER stress modulator salubrinal (Sal) could stop apoptosis and restore normal function to dysfunctional mitochondria. Logsdon et al. (2014) also investigated administration of Sal for the treatment of TBI and the associated neuropsychiatric symptoms. Logsdon et al. (2014, p. 12) also concluded that ER stress regulation *via* Sal was capable of reducing apoptosis while also limiting impulse-like behavior in rats afflicted with TBI.

Glial cell activation remains a common factor in secondary neurodegeneration following the initial injury in both TBI and TON (Rodgers et al., 2012; Bond and Rex, 2014; Choi et al., 2014; Tao et al., 2017; Evanson et al., 2018; DeJulius et al., 2021; Hetzer et al., 2021; Zhou et al., 2021; Priester et al., 2022). As previously stated, after injury the BBB/BRB can break down, allowing for systemic macrophages to invade the CNS and take on an active microglial morphology (Bond and Rex, 2014; Brooks et al., 2014; McMenamin et al., 2019; DeJulius et al., 2021). Injury also activates the resting microglia cells, which then activate the neuroinflammatory response (Bond and Rex, 2014; DeJulius et al., 2021; Zhou et al., 2021; Priester et al., 2022). Activated glial cells will release proinflammatory cytokines and phagocytose cells; however, they do this while releasing large amounts of ROS (Dröge, 2002; Bond and Rex, 2014; DeJulius et al., 2021). In an overall healthy environment, this neuroinflammatory response serves to heal tissue, but when the level of ROS begins to outcompete the cells’ ability to produce antioxidants this chain of events can lead to neural degeneration (Bond and Rex, 2014; Priester et al., 2022). One study determined the BBB was disrupted as early as 30 min after blast-induced TBI (Logsdon et al., 2014). They also observed astrocyte activation after an increase in ER stress and subsequent upregulation of UPR (Logsdon et al., 2014). Logsdon et al. (2014, p. 7) noted the activation of astrocytes is characteristic of neuroinflammation and cell death. Studies examining astrocyte activation tend to utilize glial fibrillary acidic protein (GFAP) (Choi et al., 2014; Evanson et al., 2018). GFAP immunoreactivity was increased after TBI in both Choi et al. (2014), Evanson et al. (2018). Interestingly Evanson et al. (2018) observed an increase in size of microglial cells 24 h after injury in the optic track only, but at 24 h after injury they did not observe increased GFAP immunoreactivity (Evanson et al., 2018). This would indicate a shape change in microglia occur prior to increased astrocyte activation. At 7 days post injury the microglial cells were still increased in size, but only in the optic tract, while GFAP immunoreactivity was found to be increased at this timepoint in the optic tract, LGN, and SC (Evanson et al., 2018). A separate study determined GFAP immunoreactivity was increased in both the retina and optic nerve after single and repeated blast overpressure induced trauma (Choi et al., 2014). It may be that glial cell activation plays an important role in the accumulation of ROS and subsequent neurodegeneration observed in both the brain and retina following traumatic injury. The infiltration of systemic macrophages, upon the disruption of the BBB/BRB, in addition to the native CNS activated glial cells may overwhelm the antioxidant systems in place for normal glial cell response (Zhou et al., 2021). This may ultimately lead to neurodegeneration after injury.

## 4. Current therapeutic approaches

There are many different therapeutic studies being conducted targeting not only antioxidant mechanisms, but also other elements in the injury and neurodegenerative cascade. One such promising therapeutic is tauroursodeoxycholic acid (TUDCA). TUDCA is comprised of taurine, which is a common amino acid in the retina that retinal cells need to uptake for cellular function (Daruich et al., 2019). TUDCA has been shown to have anti-inflammatory, anti-apoptotic, antioxidant, and neuroprotective effects (Elia et al., 2015; Gómez-Vicente et al., 2015; Daruich et al., 2019). TUDCA was observed to promote RGC survival after optic nerve crush in rats and has also been utilized in several studies for the treatment of neurodegenerative disorders such as ALS, PD, AD, and HD (Vang et al., 2014; Elia et al., 2015; Daruich et al., 2019; Kitamura et al., 2019). TUDCA has been observed to reduce ROS, limit microglial cell activation, reduce ER stress, and suppress inflammatory processes (Vang et al., 2014; Gómez-Vicente et al., 2015; Daruich et al., 2019). It has also been reported that TUDCA can preserve the BRB (Daruich et al., 2019).

Another promising therapeutic is ibudilast, which is a cAMP phosphodiesterase (PDE) inhibitor (Cueva Vargas et al., 2016). Ibudilast works to attenuate ROS production by inhibiting phosphodiesterase which in turn increases cAMP levels (Tahvilian et al., 2019). The increase of cAMP suppresses the expression of sigma (σ) receptors, which can be responsible for increasing ROS (Ostenfeld et al., 2005; Tahvilian et al., 2019). In addition, ibudilast reduces glial cell activation and subsequently reduces pro-inflammatory cytokines (Lee et al., 2012; Cueva Vargas et al., 2016). Ibudilast is known to suppress the proinflammatory protein macrophage migration inhibitory factor (MIF), while also increasing the concentration of anti-inflammatory cytokines and neurotrophic factors (Cho et al., 2010; Lee et al., 2012). Like TUDCA, ibudilast has been investigated in neurodegenerative diseases such as MS and ALS (Fox et al., 2018; Oskarsson et al., 2021). Rodgers et al. (2012) examined the connection between post-injury neuroinflammation and post-traumatic anxiety in a TBI rat model. In this study they utilized OX-42 to determine microglia activation and GFAP to determine astrocyte activation (Rodgers et al., 2012). They observed increased labeling for both microglia and astrocytes after injury (Rodgers et al., 2012). Rodgers et al. (2012) utilized ibudilast and determined ibudilast was capable of reducing reactive gliosis while also attenuating anxiety behaviors in post-TBI rats (Rodgers et al., 2012). Based on these studies, ibudilast may be a promising therapeutic toward the reduction of microglial and astrocyte activation and subsequent reduction of ROS production.

Erythropoietin (EPO) is another potential therapeutic that may be able to reduce elevated ROS in neurodegenerative cells (Bond and Rex, 2014). EPO can limit the immune cell response and migration, and thus may be beneficial in attenuating the elevation of ROS and proinflammatory cytokines through this process (Bond and Rex, 2014). EPO has been utilized in a TON model to reduce ROS (DeJulius et al., 2021). In this study, they determined sustained release of EPO *via* poly(propylene sulfide) (PPS) and poly(lactic-co-glycolic acid) (PLGA) microspheres resulted in neuroprotective properties after iTON (DeJulius et al., 2021). EPO has also been examined for neuroprotective effects in a mouse model of Parkinson’s Disease (Dhanushkodi et al., 2012). In this study they determined EPO may result in increased axonal sprouting and neuroprotective effects in PD (Dhanushkodi et al., 2012).

Researchers have begun analyzing whether attenuation of pro-inflammatory cytokines such as tumor necrosis factor (TNF) can result in neuroprotective effects. TNF concentrations are elevated after traumatic injury and in several neurodegenerative diseases such as AD, MS, and PD (Fontaine et al., 2002). TNF is one of the most important proinflammatory cytokines that is upregulated after TON (Tse et al., 2018). TNF helps to regulate immune cell function and plays a pivotal role in the pathogenesis of almost all neurodegenerative diseases (Tse et al., 2018; Chen et al., 2022). TNF-α has been observed to increase ROS through the activation of cyclin-dependent kinase 5 (Cdk5) (Sandoval et al., 2018). TNF’s ability to increase ROS may be why it is found to play a role in so many neurodegenerative diseases. Fontaine et al. (2002) analyzed whether the upregulation of the molecule TNF was causing the degeneration or whether which TNF receptor the molecule bound to was causing the degeneration. They determined TNF Receptor 2 (TNF-R2) may promote neuroprotection while TNF Receptor 1 (TNF-R1) may promote neurodegeneration (Fontaine et al., 2002). In a healthy environment, the rate of activity between the two receptors should be relatively balanced, but if TNF-R2 were to become overactive then neurodegeneration could occur (Fontaine et al., 2002). Fontaine et al. (2002) tried blocking the TNF molecule itself but observed no significant difference in cell death or survival. When they created a TNF-R2 deficit they observed an increase in retinal cell death *via* TNF; however, when they created a deficit in TNF-R1 they observed a neuroprotective response from TNF (Fontaine et al., 2002). Since TNF is upregulated in many ocular disorders and neurodegenerative diseases, it may be beneficial to attenuate TNF levels and TNF-R1 activity as a means to promote neuroprotection.

Other researchers have focused on replacing the abnormal mitochondria. As previously explained, mitochondria are a major source of ROS production and a site for ROS damage. In a rodent model one group was able to transfer isolated and normally functioning mitochondria into neuronal schizophrenia cells (Robicsek et al., 2018). The addition of normal mitochondria reduced the neurological deficits associated with schizophrenia in their rodent model (Robicsek et al., 2018). This technique has also been applied to the ocular environment by Nascimento-dos-Santos et al. (2020) in their optic nerve injury model. In this study, liver mitochondria were transplanted into the retina and were successful in regulating the oxidative metabolism of these cells (Nascimento-dos-Santos et al., 2020). They observed successful protection of RGCs and an increase amount of optic nerve axons with the transplanted mitochondria (Nascimento-dos-Santos et al., 2020).

## 5. Discussion

The brain and retina are uniquely connected, which allows for the ocular environment to serve as a model for CNS degeneration and disease. In addition, traumatic brain injury can often result in disturbances or total loss to the visual system (Sarkies, 2004; DeJulius et al., 2021). Trauma nurses are even tasked with checking a patient’s pupillary reflex when TBI is suspected (Adoni and McNett, 2007). The unique relationship between the retina and the brain allows us to glean neurodegenerative processes in an accessible environment (Cheung et al., 2017; Bernardo-Colón et al., 2018). As described in this paper, much can be learned about the cellular response to traumatic brain injury through examination of the retina after injury. Both TBI and TON experience secondary injury *via* neuroinflammatory responses that can occur several hours to days after injury. Treatment protocols must be determined to suppress harmful over production of ROS, and up regulation of glial cells, proinflammatory cytokines, and other cellular elements responsible for increased neurodegeneration in the unbalanced system. Current treatment options are limited and have had minimal success. Determination of why these treatments fail will allow researchers to determine not only the maximal therapeutic intervention but will also allow researchers to better understand the disease progression.

### 5.1. Why might antioxidant treatments fail?

It is clear that ROS plays a pivotal role in neurodegeneration in both the brain and retina. Thus, it would make sense to limit the concentration of ROS *via* antioxidants for preservation of the neurological system from degeneration. Many have tried this approach, but unfortunately most antioxidant therapeutics tend to fail during clinical trials (Priester et al., 2022). The question then becomes why do antioxidant therapeutics fail if ROS play such a major role in neurodegeneration? Dröge (2002) states that utilizing large concentrations of antioxidants to combat ROS may actually be damaging as the body does need some small concentrations of ROS to continue normal cellular functions and processes. It may be excessive use of antioxidants end up resulting in no change to neurodegeneration because they are over limiting ROS, and the homeostatic balance has shifted in the opposite direction. Grotegut et al. (2020) examined minocycline’s ability to reduce inflammation in a retinal degeneration model. Minocycline acts as a microglial inhibitor, which can lower ROS by suppressing the amount of active microglia (Grotegut et al., 2020). In their study they concluded that the low dose of minocycline resulted in better neuroprotection and suppression of inflammatory and apoptotic processes than the higher dosage (Grotegut et al., 2020). On the other hand, Schnichels et al. (2021) examined cyclosporine A’s ability to protect RGCs after hypoxia. In this study, they did not observe a significant protective effect for RGCs when a 6 μg/mL dosage was utilized (Schnichels et al., 2021). They did observe a significant protective effect when the higher dosage, 9 μg/mL, was utilized (Schnichels et al., 2021). To achieve proper neuroprotection from ROS *via* antioxidant therapeutics it is necessary to fully understand the degenerative processes occurring during trauma and disease mediated neurodegeneration. The means in which we can treat these disorders depends on the ways ROS interacts with the cellular environment to exacerbate damage. In some instances, it may be necessary to utilize lower doses rather than flood the CNS with antioxidant therapeutics. The means in which each therapeutic acts upon the CNS must also be established in order to determine the dosage required to obtain neuroprotective properties.

Determining the dosage dependent manner of antioxidant therapeutics is only one piece of the puzzle. Neurodegeneration through ROS is multifaceted and includes elements such as BBB/BRB breakdown, infiltration of systemic macrophages, activation of glial cells, mitochondrial dysfunction, and redox imbalance. The struggle of treating neurodegeneration after injury is determining which section of the injury process needs attention. In order to answer this question, it may depend on the timing of therapeutic intervention. Tao et al. (2017) determined ROS elevation started to rise around 30 min after sonication-induced TON. Logsdon et al. (2014) reported BBB breakdown around 30 min after TBI and then increased mRNA stress response genes at 3 h after injury. They also noted DNA damage-inducible protein 34 (GADD34) was upregulated at 24 h after injury with astrocyte activation occurring afterward (Logsdon et al., 2014). Deng et al. (2007) also noted an increase in oxidative stress markers around 30 min after TBI with these levels remaining elevated for 3–6 h before returning to control levels. It may be that in order for antioxidant therapeutics to work, they need to be administered as close to the injury event as possible. If therapeutic intervention is delayed, the ROS elevation event may have already created substantial damage and the body may have moved toward activation of other cellular cascades that result in further neurodegeneration, such as activation of astrocytes. From the research presented, it appears ROS elevation and oxidative stress is one of the first mechanisms to occur after the injury event; however, due to polytrauma and the fact that noticeable vision loss does not always occur immediately after injury, patients with TON/TBI may not seek treatment until well after this crucial window of time. Therefore, the therapeutic intervention utilized must take into account the injury timeline and how the secondary neurodegenerative cascade has progressed. In some instances, ROS reductive treatments may be ineffective if the injury process has moved downstream; therefore, targeting other cellular cascades in addition to the ROS reductive therapeutics in a mixed approach may be the best course of action when treatment after injury has been delayed.

Further research is needed to determine the best approach to mitigate neurodegenerative processes in the CNS. In traumatic injury the BBB/BRB has been observed to breakdown, this breakdown may actually help facilitate the delivery of therapeutic intervention methods such as antioxidants as some antioxidant therapeutics cannot cross the BBB/BRB under normal physiological conditions (Singh et al., 2019). In other neurodegenerative disorders, this breakdown may not occur, or may occur at a different time points after the beginning of neurodegeneration. Additional research is also warranted to further determine the pathophysiological time course of both traumatic and genetic forms of neurodegeneration. The determination of this time course may allow researchers to develop efficacious treatment protocols for individuals afflicted with neurodegenerative processes in both the brain and retina.

## Author contributions

AR and WR drafted the manuscript. MR edited the manuscript. All authors contributed to the literature review and discussions and approved the submitted version.
